# Supplementary material for: Oncogenic PIK3CA induces centrosome amplification and tolerance to genome doubling
Source: Nat Commun. 2017 Nov 24;8:1773. doi: 10.1038/s41467-017-02002-4 (PMC5701070; doi:10.1038/s41467-017-02002-4)
Supplement: Supplementary file 1 — Supplementary Information [file 41467_2017_2002_MOESM1_ESM.pdf]

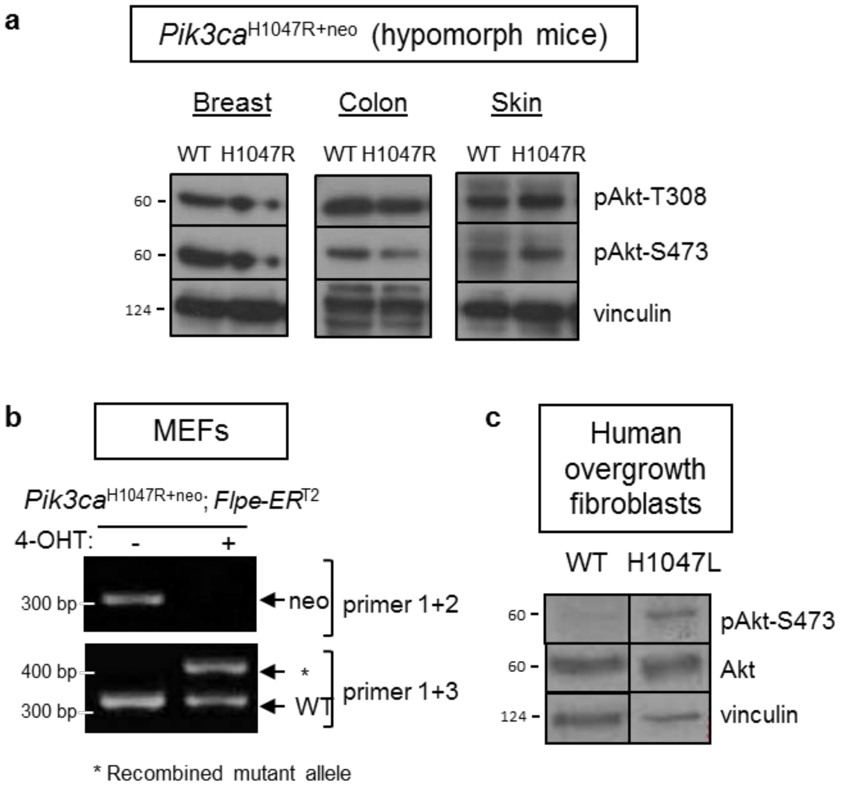

Supplementary Figure 1: Characterisation of hypomorph *Pik3ca*$^{\text{H1047R+neo}}$ mice and cells, and *PIK3CA*-WT and -mutant human fibroblasts. a) No increased Akt phosphorylation in tissues from WT and hypomorph *Pik3ca*$^{\text{H1047R+neo}}$ mice. **b**) Results of PCRs in *Pik3ca*$^{\text{H1047R+neo}}$;*Flpe-ER*$^{\text{T2}}$ MEFs before (top panel, presence of *neo* selection cassette) or after treatment with 4-OHT (lower panel, recombined allele). Primers 1 and 2 (shown in Fig. 1a) amplify a 307-bp fragment that reveals the presence of the *neo* selection cassette. Primers 1 and 3 amplify a 340-bp fragment in the *Pik3ca*^{WT} allele and a 425-bp fragment in the *Pik3ca*-targeted allele that contains the vestige *frt* site left after Flp-mediated recombination of the *neo* selection cassette. **c**) Phosphorylation levels of Akt in human fibroblasts derived from the arm (*PIK3CA*^{WT}) or leg (*PIK3CA*$^{\text{H1047L}}$) from a human subject with mosaic mutant *PIK3CA* expression.

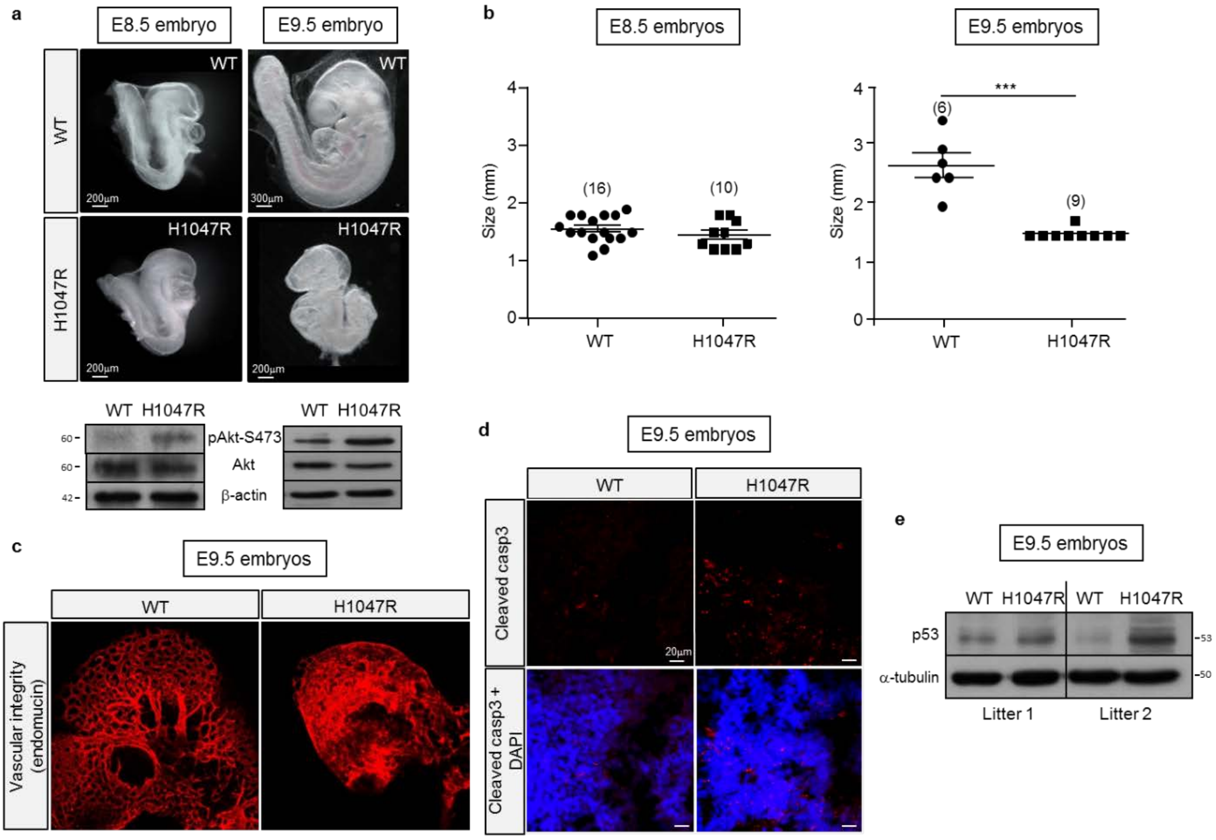

Supplementary Figure 2: Characterization of *Pik3ca*^{H1047R} embryos. a) Representative images of E8.5 and E9.5 *Pik3ca*^{WT} and *Pik3ca*^{H1047R} embryos. Western blots underneath show Akt phosphorylation in whole embryo extracts. **b**) Size of E8.5 and E9.5 *Pik3ca*^{WT} and *Pik3ca*^{H1047R} embryos. Statistically significant differences are indicated by *** ($P < 0.001$), as determined by the non-parametric Mann-Whitney *t*-test (two-tailed). Numbers between brackets denote the number of embryos measured per genotype. **c**) Representative images of whole-mount E9.5 *Pik3ca*^{WT} and *Pik3ca*^{H1047R} embryos labelled with endomucin (red), illustrating the vascular plexus in the head. **d**) Apoptosis in E9.5 *Pik3ca*^{WT} and *Pik3ca*^{H1047R} embryos assessed by cleaved caspase-3 immunostaining of embryo cryosections. **e**) p53 protein levels in E9.5 *Pik3ca*^{WT} and *Pik3ca*^{H1047R} whole embryo protein extracts. Shown are cell extracts from 2 pools of embryos (3 embryos each), derived from two independent litters per genotype.

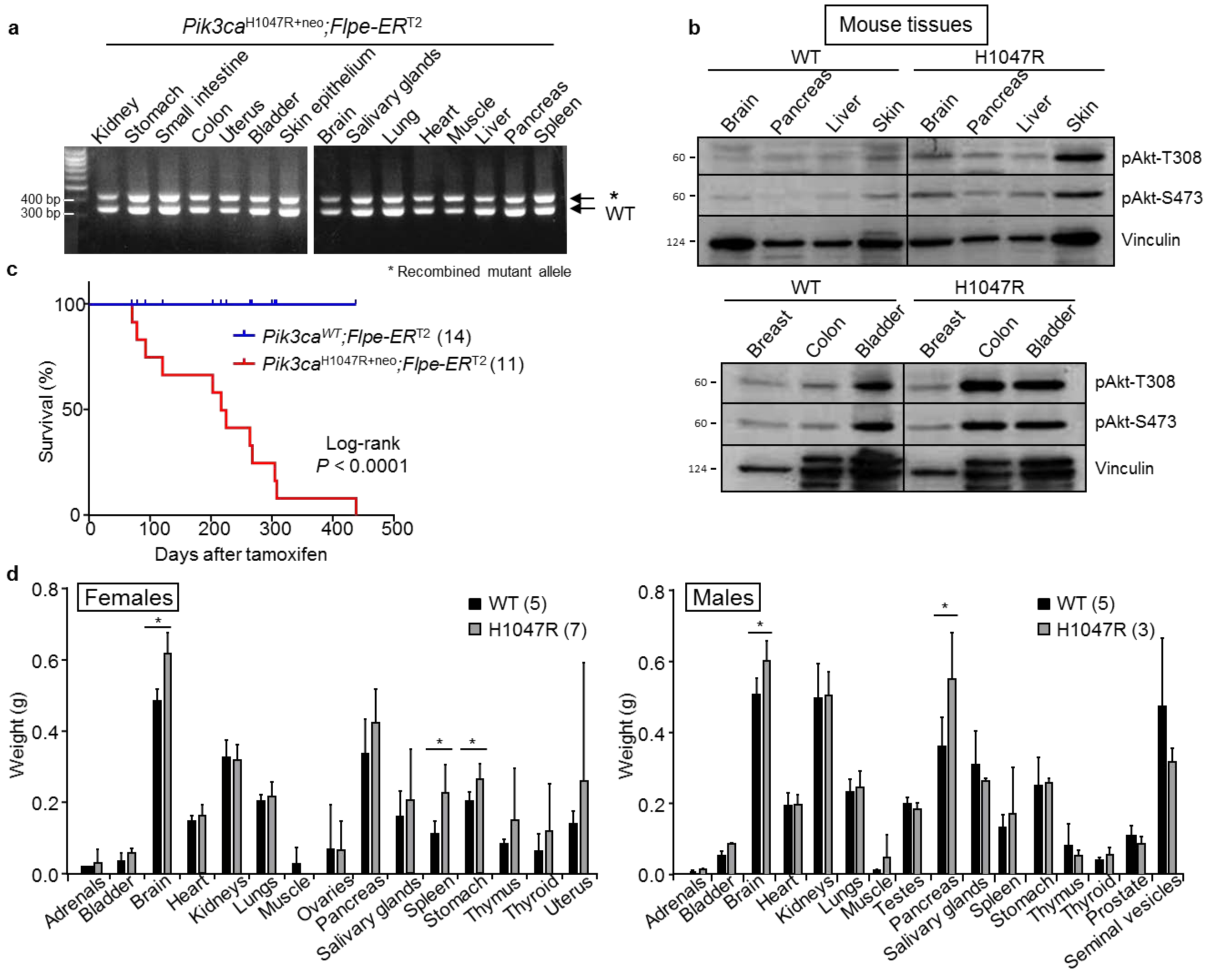

Supplementary Figure 3: **Induction of *Pik3ca*^{H1047R} in adult mice. a**) PCR analysis (explained in insert in Supplementary Figure 1) showing effective Flp-mediated recombination of the *neo* selection cassette in tissues of 8-week-old *Pik3ca*$^{H1047R+neo}$;*Flpe-ER*^{T2} mice following 5 consecutive days of *in vivo* treatment with tamoxifen (80 mgkg^{-1} per gavage). **b**) Phosphorylation of Akt in tissues of 8-week-old *Pik3ca*^{WT};*Flpe-ER*^{T2} and *Pik3ca*$^{H1047R+neo}$;*Flpe-ER*^{T2} mice following 5 consecutive days of *in vivo* treatment with tamoxifen (80 mgkg^{-1} per gavage). **c**) Survival of *Pik3ca*^{WT};*Flpe-ER*^{T2} and *Pik3ca*$^{H1047R+neo}$;*Flpe-ER*^{T2} mice following treatment with tamoxifen for 5 consecutive days (80 mgkg^{-1} per gavage) at the age of 8 weeks. *** ($P < 0.001$); log-rank (Mantel–Cox) test. Numbers in brackets denote the number of mice used per experiment. Numbers between brackets denote the number of mice used per genotype. **d**) Organ weight of *Pik3ca*^{WT};*Flpe-ER*^{T2} and *Pik3ca*$^{H1047R+neo}$;*Flpe-ER*^{T2} mice at the time of death. Statistically significant differences are indicated by * ($P < 0.05$), as determined by non-parametric Mann-Whitney *t*-test (two-tailed). Numbers between brackets denote the number of mice used per genotype.

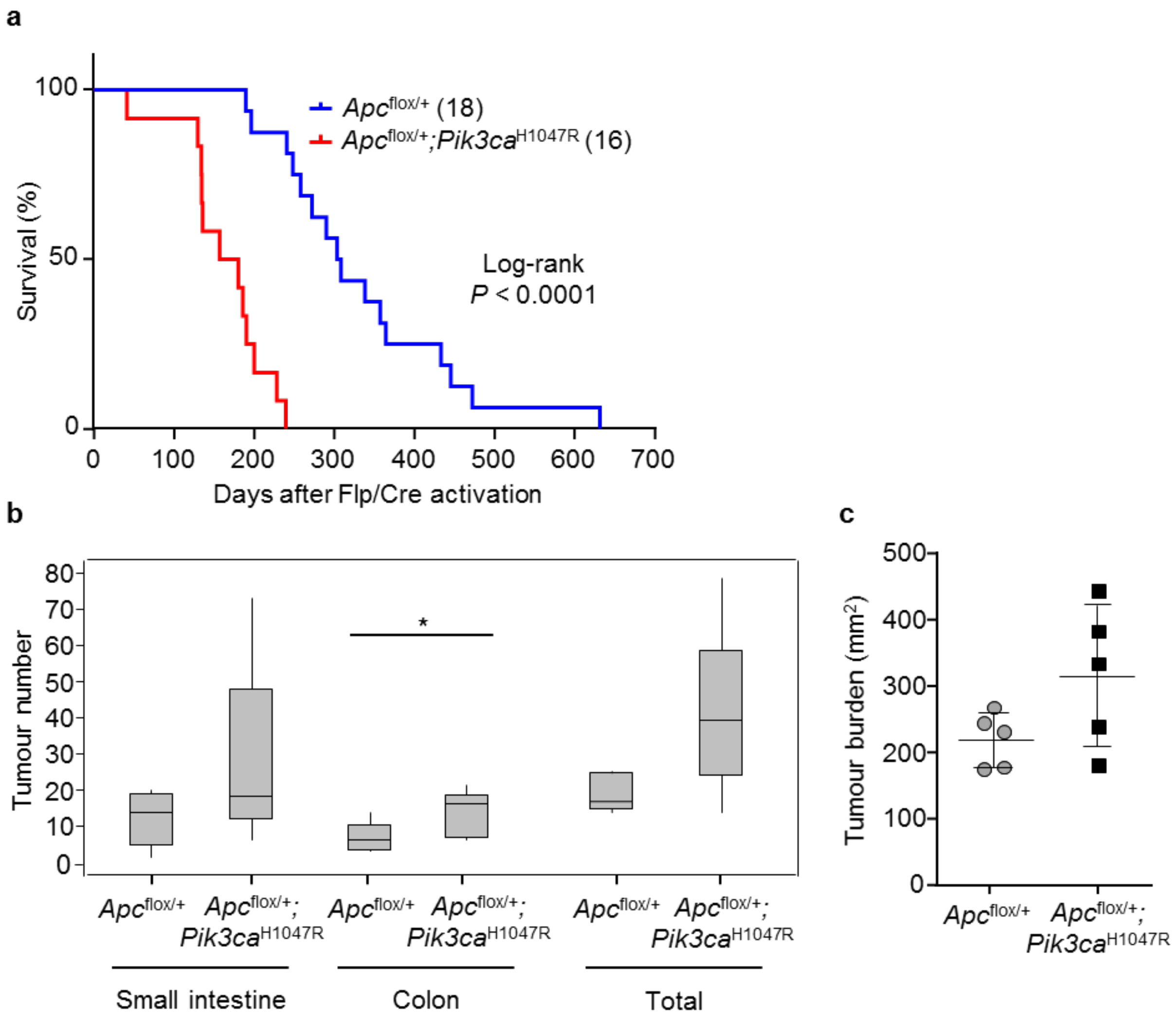

Supplementary Figure 4: *Pik3ca*$^{\text{H1047R}}$ activation on an Apc$^{\text{flox/+}}$ background accelerates tumour development. a) Kaplan–Meier survival curves of *Apc*$^{\text{flox/+}}$ and *Apc*$^{\text{flox/+}}$;*Pik3ca*$^{\text{H1047R}}$ mice. * ($P < 0.05$), *** ($P < 0.001$); log-rank (Mantel–Cox) test. Numbers in brackets denote the number of mice used per experiment. **b**) Box plots showing the tumour number in the small intestine , colon and in both (Total) in *Pik3ca*$^{\text{H1047R}}$;*Apc*$^{\text{flox/+}}$ and *Apc*$^{\text{flox/+}}$ mice (n =5 for both genotypes). * ($P < 0.05$); non-parametric Mann-Whitney *t*-test. **c**) Primary tumour burden in *Apc*$^{\text{flox/+}}$ and *Apc*$^{\text{flox/+}}$;*Pik3ca*$^{\text{H1047R}}$ mice. Tumour burden was calculated and represents the total tumour volume per mouse. (n=5 for both genotypes).

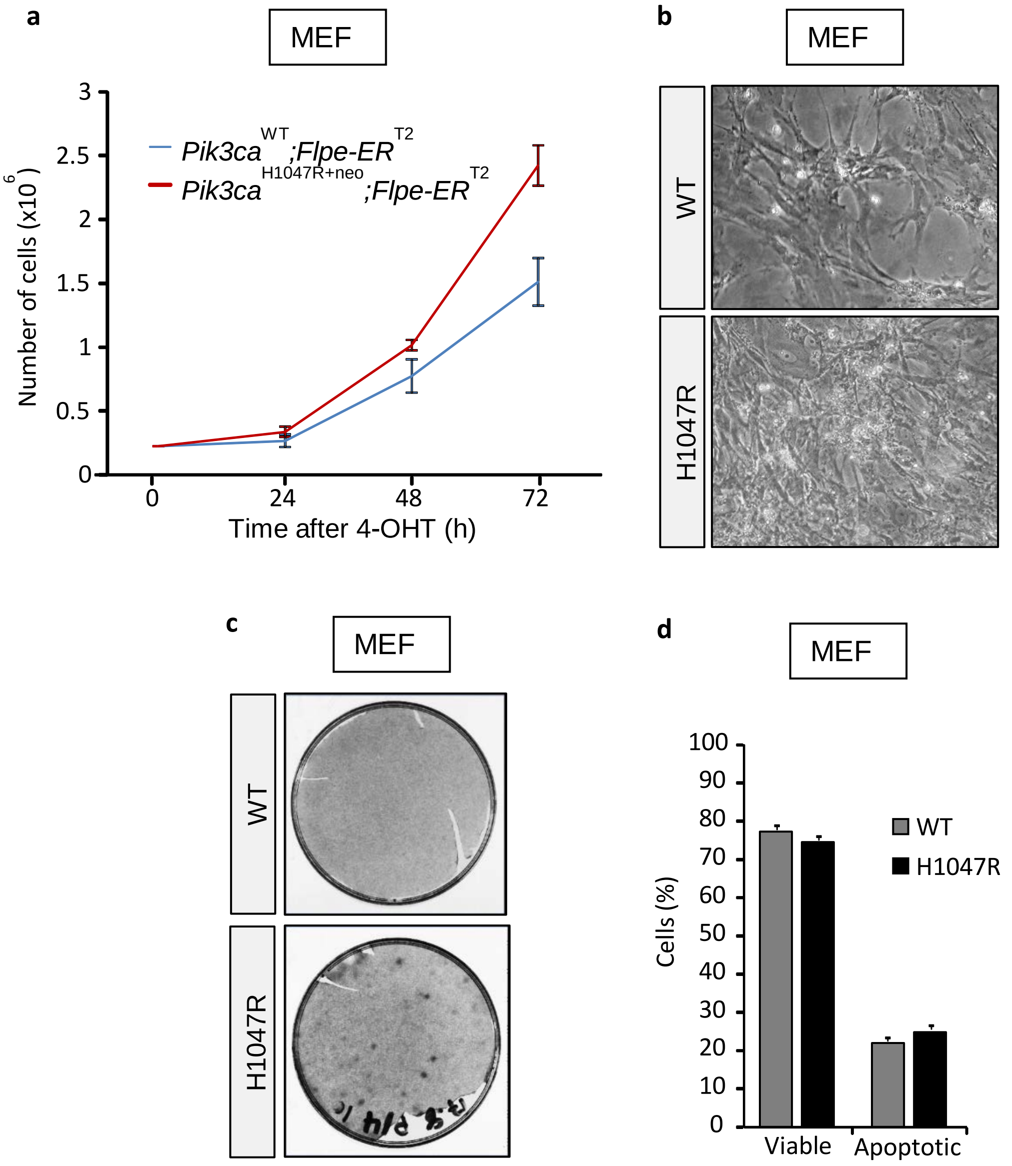

Supplementary Figure 5: Characterization of *Pik3ca*$^{H1047R+neo}$ and *Pik3ca*^{H1047R} MEFs. a) Number of viable cells, as assessed by Casy Counter cell counting, at different time points after addition of 4-OHT on day 0. 2 independent WT and 3 independent p110α^{H1047R} MEFs were analysed. Errors bars indicate SD. **b**) Representative picture of *Pik3ca*^{WT};*Flpe-ER*^{T2} and *Pik3ca*$^{H1047R+neo}$;Flpe-ER^{T2} cell cultures. **c**) Colony formation in *Pik3ca*^{WT};*Flpe-ER*^{T2} and *Pik3ca*$^{H1047R+neo}$;*Flpe-ER*^{T2} MEFs. **d**) Frequency of viable and apoptotic cells 5 days after addition of 4-OHT in E13.5 *Pik3ca*^{WT};*Flpe-ER*^{T2} and *Pik3ca*$^{H1047R+neo}$;*Flpe-ER*^{T2} MEFs assessed by Annexin V and PI staining. 20,000 cells were acquired from 8 independent WT and 8 independent p110α^{H1047R} MEFs. Errors bars indicate SD.

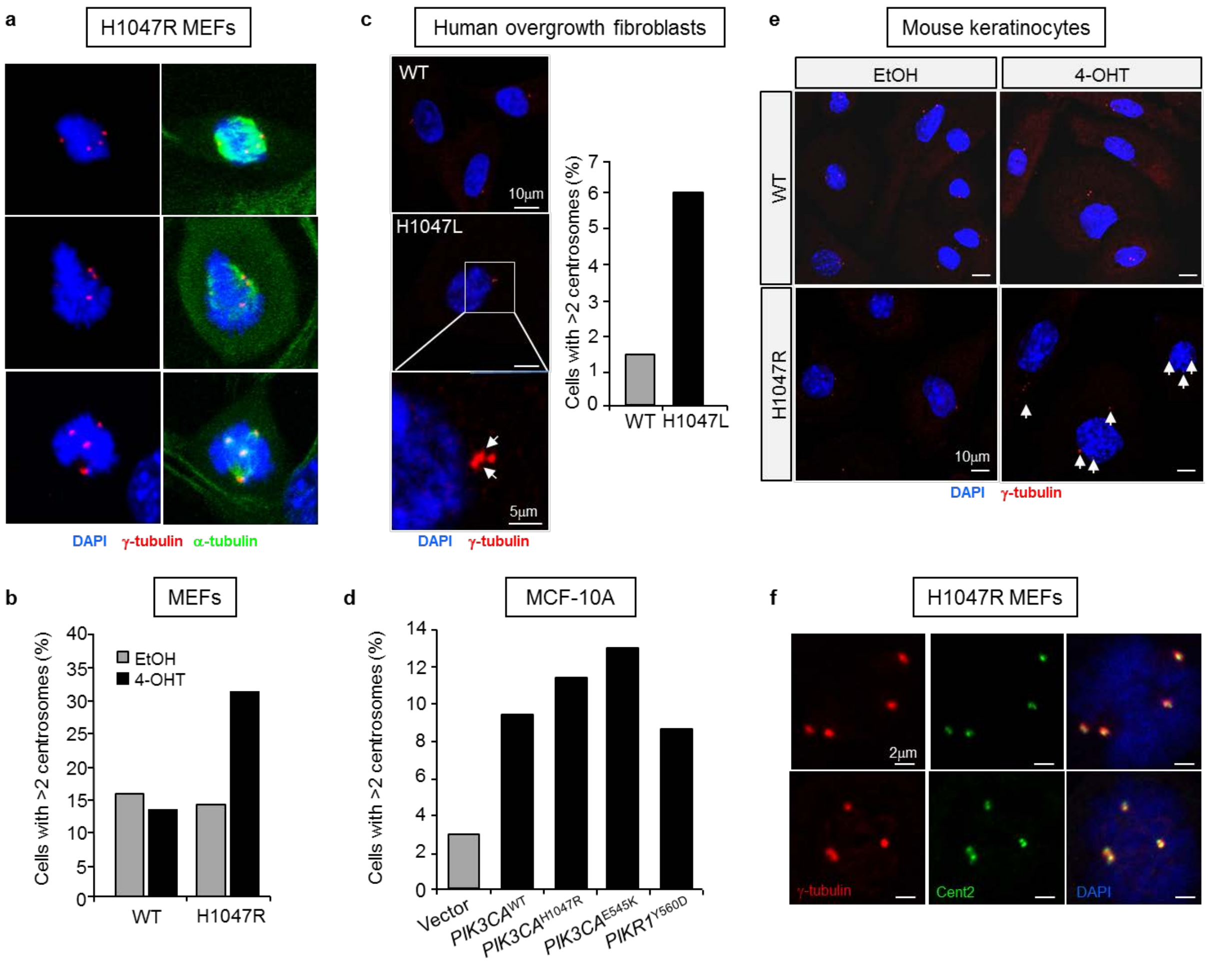

Supplementary Figure 6: Centrosome amplification upon p110α^{H1047R} expression. a) Representative immunofluorescence images of mitotic spindle conformations observed in *Pik3ca*$^{H1047R+neo}$;*Flpe-ER*^{T2} MEFs. Centrosomes were stained with anti-γ-tubulin, microtubules with anti-α-tubulin and DNA with DAPI. **b**) Frequency of cells with centrosome amplification in *Pik3ca*^{WT};*Flpe-ER*^{T2} and *Pik3ca*$^{H1047R+neo}$;*Flpe-ER*^{T2} MEFs treated for 3 days with 4-OHT or vehicle. Centrosomes were counted in anti-γ-tubulin-stained cells, scoring 200 cells per genotype and condition. **c**) Representative immunofluorescence image of centrosome amplification in *PIK3CA*^{H1047L} human dermal fibroblasts. Centrosomes were stained with anti-γ-tubulin and DNA with DAPI. Graph shows the frequency of cells with centrosome amplification. 500 cells were scored per genotype. d) Frequency of cells with centrosome amplification in MCF-10A breast epithelial cells transfected with a *PIK3CA*^{WT}, *PIK3CA*^{H1047R}, *PIK3CA*^{E545K}, *PIKR1*^{D560Y} expression vector or control (empty) expression vector. 300-400 cells were scored per condition **e**) Representative immunofluorescence images of centrosome amplification in *Pik3ca*^{WT};*Flpe-ER*^{T2} and *Pik3ca*$^{H1047R+neo}$;*Flpe-ER*^{T2} keratinocytes treated for 2 days with 4-OHT or vehicle. **f**) Representative immunofluorescence images showing extra centrosomes in a p110α^{H1047R} MEF cell composed of two centrioles.

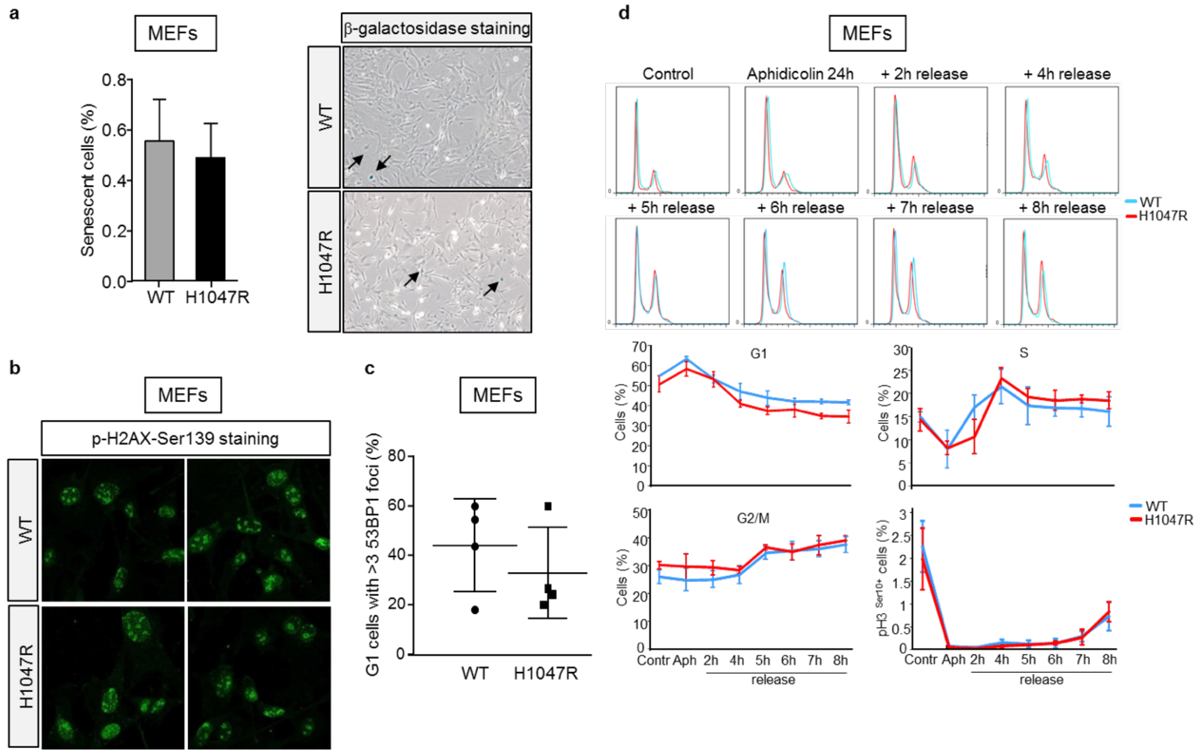

Supplementary Figure 7: Activation of p110α^{H1047R} in MEFs does not induce senescence, DNA damage or cell cycle alterations. a) Frequency of senescent cells in *Pik3ca*^{WT};*Flpe-ER*^{T2} and *Pik3ca*$^{H1047R+neo}$;*Flpe-ER*^{T2} MEFs, assessed by β-galactosidase staining. Representative images are shown with arrows pointing towards senescent cells. 3 independent WT and 3 independent p110α^{H1047R} MEFs were analysed. Error bars indicate SEM. **b**) Staining of γH2AX foci in *Pik3ca*^{WT};*Flpe-ER*^{T2} and *Pik3ca*$^{H1047R+neo}$;*Flpe-ER*^{T2} MEFs, 3 days after treatment with 4-OHT. **c**) Frequency of G1 phase nuclear bodies containing p53-binding protein 1 (53BP1) in *Pik3ca*^{WT};*Flpe-ER*^{T2} and *Pik3ca*$^{H1047R+neo}$;*Flpe-ER*^{T2} MEFs, 3 days after treatment with 4-OHT. 4 independent WT and 4 independent p110α^{H1047R} MEFs were analysed. Error bars indicate SD. **d**) Time-course cell cycle analysis of aphidicolin-synchronized MEFs cultures by PI staining. Mitotic cells were determined by phospho-Histone-3^{Ser10} staining. 3 independent WT and 3 independent p110α^{H1047R} MEFs were analysed. Error bars indicate SD.

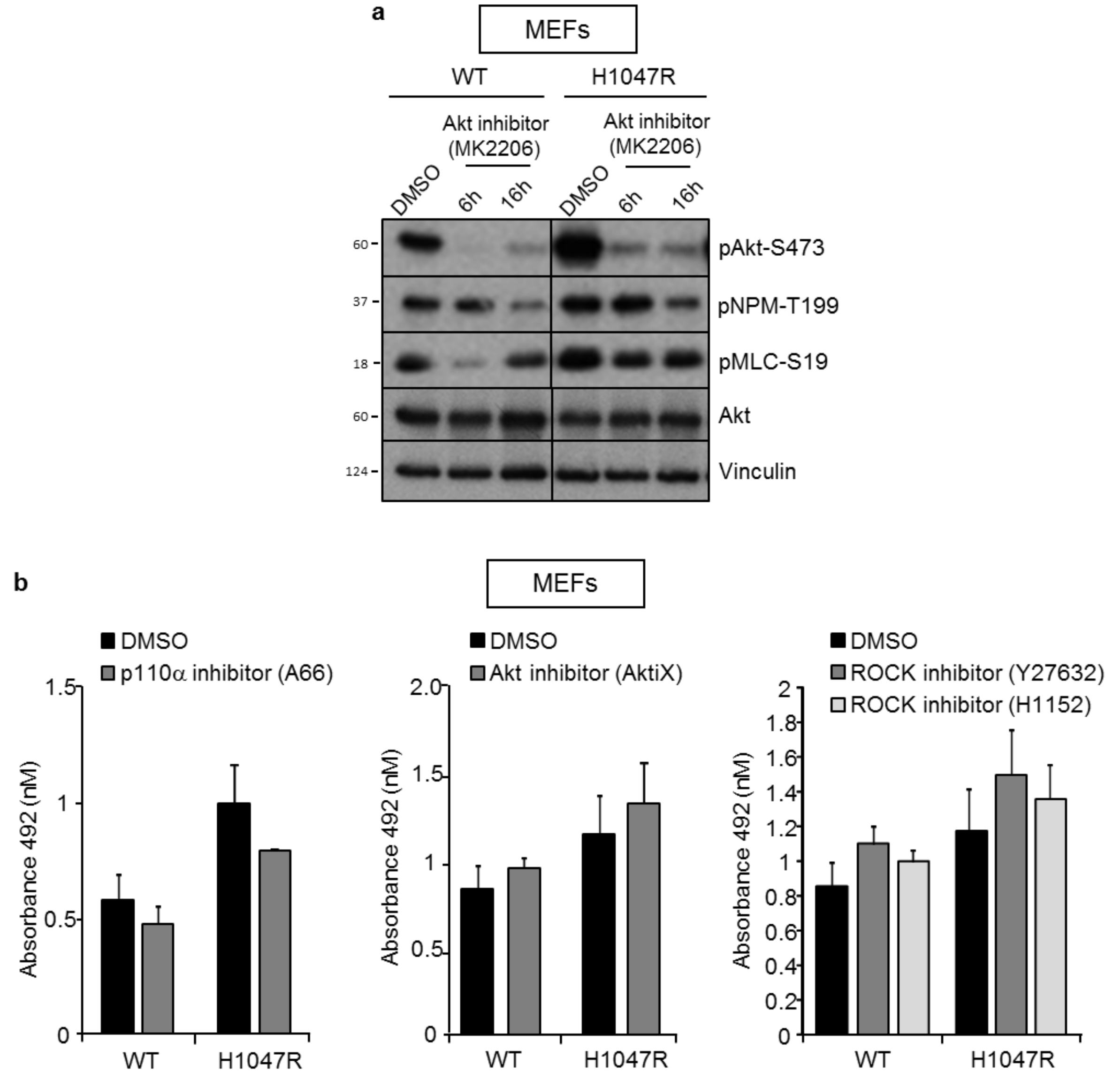

Supplementary Figure 8: Impact of Akt inhibition on signalling towards centrosome duplication and of inhibition of the indicated PI3K pathway inhibitors on MEF proliferation. a) Effect of AKT inhibition (by 1 µM MK2206) on signalling pathways involved in centrosome duplication. 3 independent *Pik3ca*^{WT};*Flpe-ER*^{T2} and 3 independent *Pik3ca*$^{H1047R+neo}$;*Flpe-ER*^{T2} MEFs were analysed. A representative experiment is shown. **b**) Impact of the inhibition of p110α (by 3 µM A66), Akt (by 1 µM Akti X) or ROCK (by 10 µM Y27632 or 0.5 µM H1152) on the proliferation of MEFs assessed by MTS. Cells were treated for 3 days with the indicated compounds. Error bars indicate standard deviation of 2 independent experiments using 2 independent MEF cell lines per genotype.

a

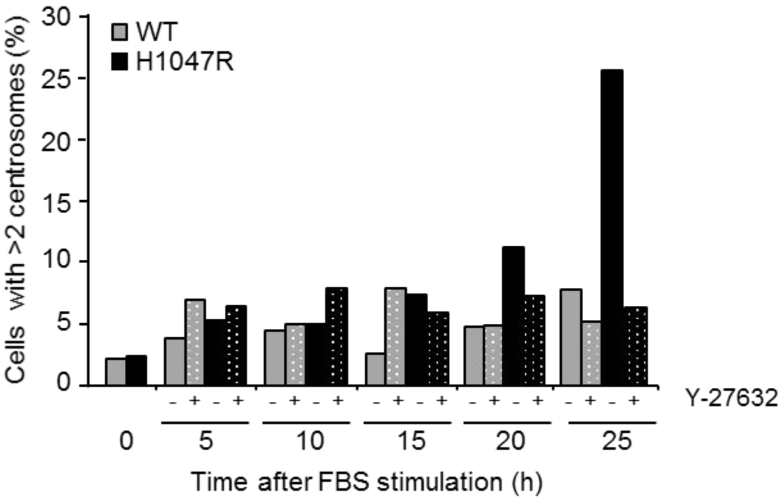

b

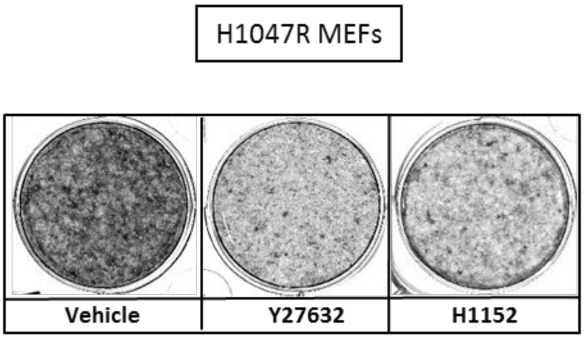

Supplementary Figure 9: Impact of ROCK inhibition on centrosome amplification before cytokinesis and on cell transformation in primary p110α^{H1047R} MEFs. **a**) Centrosome number during the first cell cycle upon induction of p110α^{H1047R} expression (200 cells per genotype and time point were scored for the centrosome analysis) in the presence of the ROCK inhibitor Y27632 (10 μM). **b**) Focus formation assay on early passage primary p110α^{H1047R} MEFs treated with vehicle or the ROCK inhibitors Y27632 (10 μM) or H1152 (0.5 μM) for 10 days.

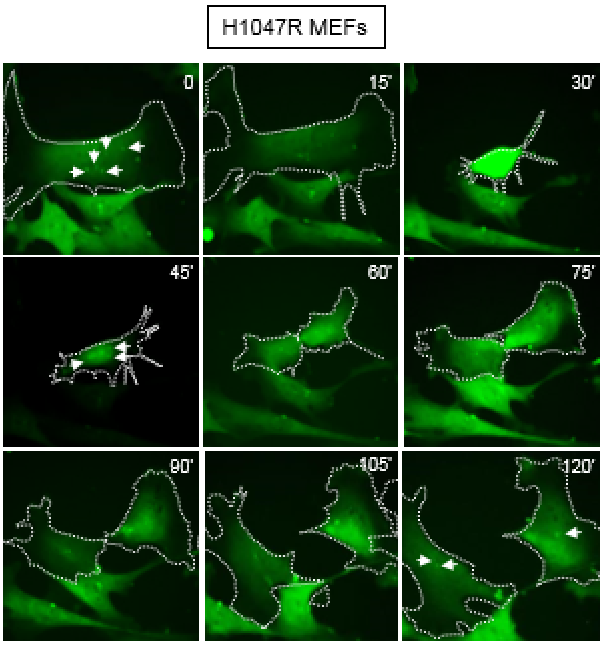

Supplementary Figure 10: Centrosome clustering in the presence of p110α^{H1047R}. Time-lapse microscopy images of a Cent2-GFP;*Pik3ca*$^{H1047R+neo}$;*Flpe-ER*^{T2} MEF cell with centrosome amplification, which is going through mitosis. White arrows point to centrosomes. Dashed lines contour individual cells. Images were taken every 15 min.

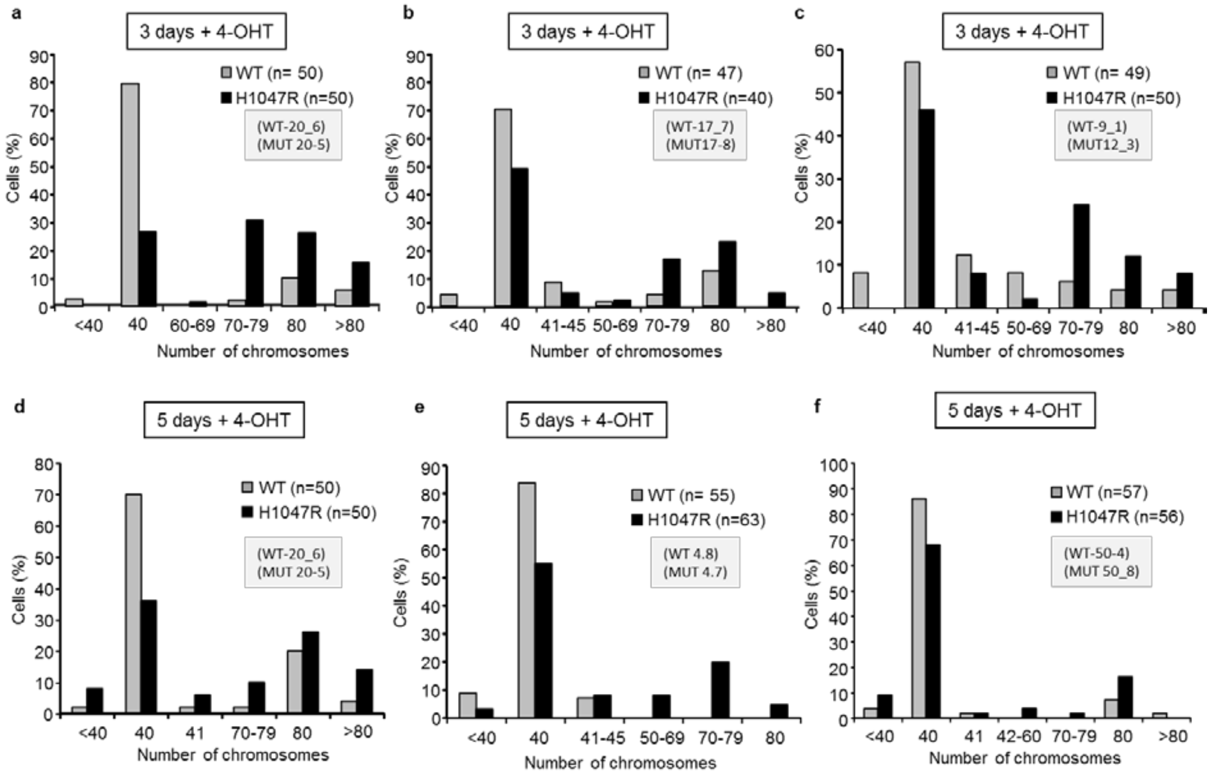

Supplementary Figure 11: p110α^{H1047R} expression leads to aneuploidy in MEFs. (**a-f**) Analysis of chromosome numbers in WT and p110α^{H1047R} MEF pairs, 3 or 5 days after treatment with 4-OHT. In a) and d), the same pair of MEFs was analysed on day 3 and day 5. In b), c), e) and f), independent MEF pairs were analysed. The names of the individual MEF clones used are indicated in parentheses in grey boxes. Chromosome counting was performed on DAPI-stained metaphase spreads. n is the number of spreads analysed per condition.

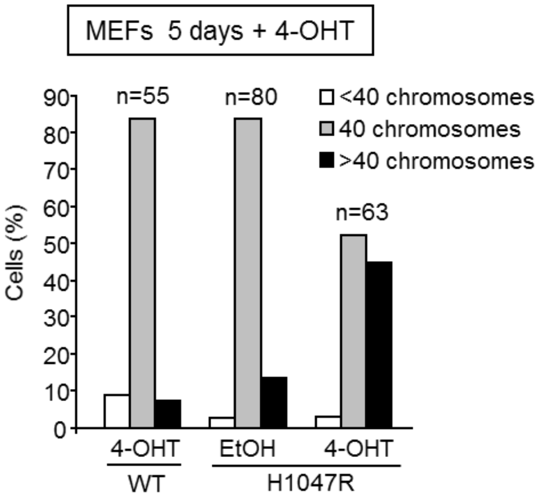

Supplementary Figure 12: Vehicle-treated p110α^{H1047R} MEFs do not show chromosome number abnormalities. Percentage of MEFs with normal ploidy (n=40) or aneuploidy (n<40 or n>40), 5 days after treatment with vehicle (EtOH) or 4-OHT. For the *Pik3ca*^{WT};*Flpe-ER*^{T2} MEFs, only the 4-OHT-treated condition is shown. Chromosome counting was performed on DAPI-stained metaphase spreads. n is the number of spreads analysed per condition.

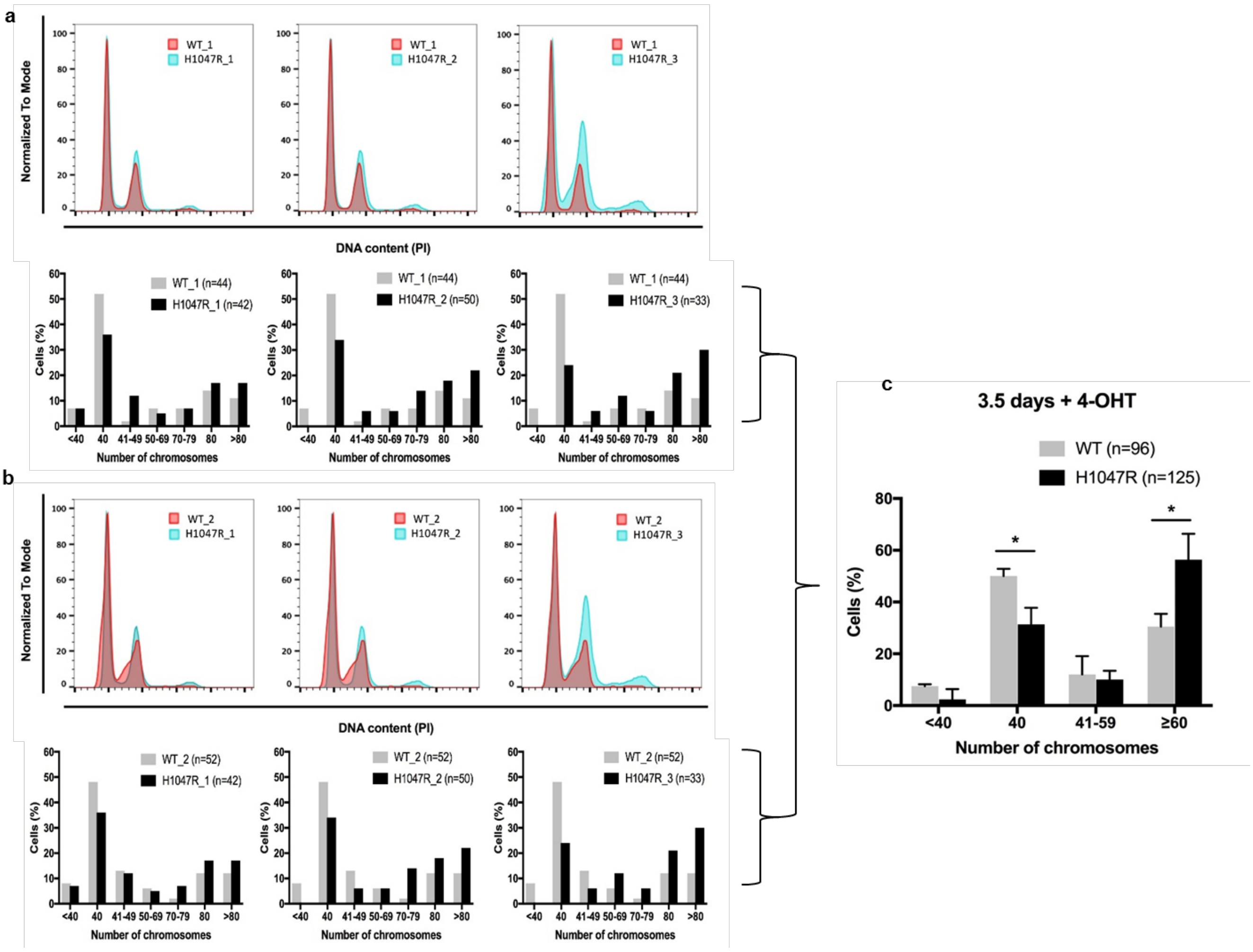

Supplementary Figure 13: Parallel FACS and metaphase spread analysis of WT and p110α^{H1047R} MEFs. Three independent p110α^{H1047R} MEF lines were analysed and compared to two independent WT MEF lines. Cells were treated with 4-OHT for 3.5 days, followed by DNA content analysis by FACS or by metaphase spread analysis. **a**,**b**) Comparison of independent p110α^{H1047R} MEF lines (H1047R_1, H1047R_2 and H1047R_3) to WT_1 and WT_2 MEF line, respectively. **c**) Grouping of chromosome numbers as determined by metaphase spreads in panels a) and b), pooling cells from 2 WT and 3 H1047R MEF lines (n= number of metaphase spread cells counted per genotype). Data are presented as the mean ± SD and statistically significant differences are indicated by * (P < 0.05), as determined by parametric unpaired *t*-test (two-tailed).

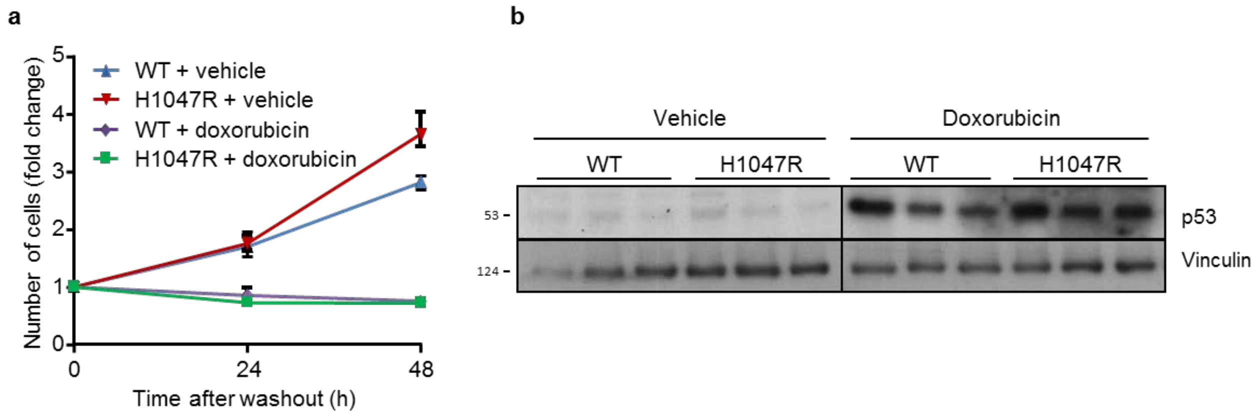

Supplementary Figure 14: Intact DNA damage checkpoint in p110α^{H1047R} MEFs. a) Impact of 20 h doxorubicin treatment on *Pik3ca*^{WT};*Flpe-ER*^{T2} and *Pik3ca*$^{H1047R+neo}$;Flpe-ER^{T2} MEFs, as assessed by live cell counting 24 h and 48 h after doxorubicin wash-out. 3 independent WT and 3 independent p110α^{H1047R} MEFs were analysed. Error bars indicate SEM. **b**) Expression levels of p53 *Pik3ca*^{WT};*Flpe-ER*^{T2} and *Pik3ca*$^{H1047R+neo}$;Flpe-ER^{T2} MEFs after 18 h treatment with doxorubicin. Lysates from 3 independent WT and 3 independent p110α^{H1047R} MEFs were analysed.

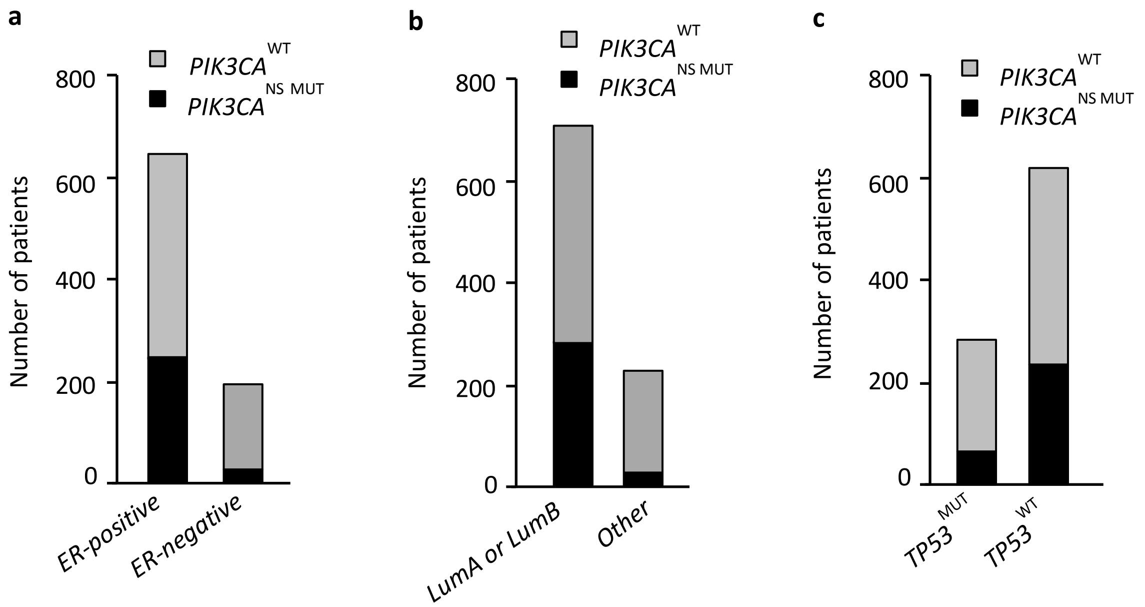

Supplementary Figure 15: Enrichment or depletion of *PIK3CA* mutations in different subgroups of breast cancer. **a**) Non-silent mutations (NS) in *PIK3CA* are enriched in ER-positive breast cancers compared to ER-negative breast cancers (P < 0.001, Fisher's exact test). **b**) Non silent mutations in *PIK3CA* are enriched in luminal breast cancers compared to other subtypes (HER-2, basal and normal-like) (P < 0.001, Fisher's exact test). **c**) Non silent mutations in *PIK3CA* show a tendency for mutual exclusivity with mutations in *TP53* (P < 0.001, Fisher's exact test).

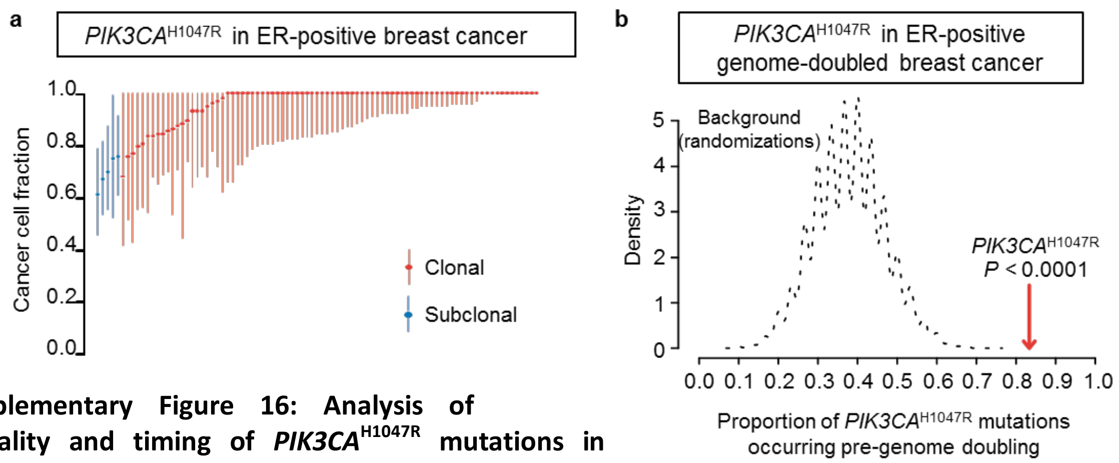

Supplementary Figure 16: Analysis of clonality and timing of *PIK3CA*$^{\text{H1047R}}$ mutations in genome-doubled ER-positive breast cancers. a) The cancer cell fraction of *PIK3CA*$^{\text{H1047R}}$ mutations in the TCGA ER-positive breast cancer cohort. Each symbol represents a non-silent somatic mutation in an individual tumour. On the basis of the probability distributions of the cancer cell fraction, mutations were determined to be either clonal (red circles, upper bound of confidence interval ≥1) or subclonal (blue circles, upper band of confidence interval <1). Error bars represent the 95% confidence interval. *PIK3CA*$^{\text{H1047R}}$ mutations are significantly more often clonal than sub clonal (P < 0.001). **b**) Timing of *PIK3CA*$^{\text{H1047R}}$ mutations in the TCGA genome-doubled ER-positive breast cancer cohort. The proportion of *PIK3CA*$^{\text{H1047R}}$ mutations that likely occurred prior to genome doubling is depicted with a dotted line representing expected distribution based on background randomizations. The graph shows that the majority of *PIK3CA*$^{\text{H1047R}}$ mutations likely precede genome doubling, more than expected by chance (P < 0.0001). Proportion pre-genome doubling is the proportion of *PIK3CA*$^{\text{H1047R}}$ mutations that occur pre genome doubling. The distribution relates to the expected number of mutations occurring pre-genome doubling. This is based on taking the same number of mutations, and randomly sampling this 10,000 times. This gives an estimate of the likelihood that a random sample of mutations would show the same number of pre-doubled mutations.

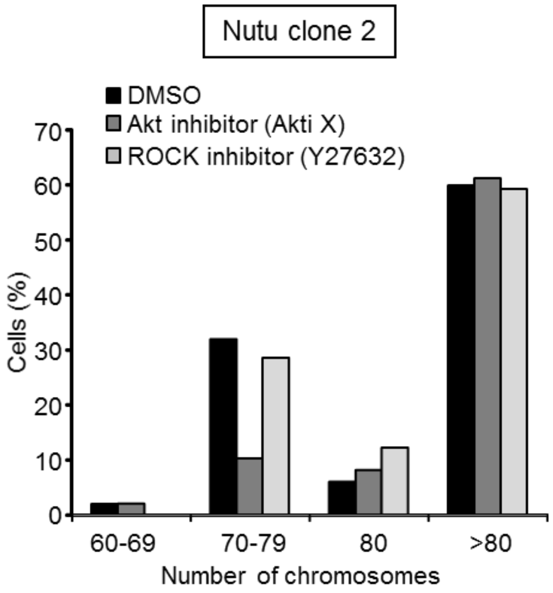

Supplementary Figure 17: Lack of an effect of selected PI3K pathway inhibitors on chromosome numbers in Nutu cells. Impact of inhibition of Akt (by 1 µM Akti X) or ROCK (by 10 µM Y27632) for 21 days on chromosome numbers of p110α^{H1047R} Nutu clone 2. Chromosome counting was performed on DAPI-stained metaphase spreads. 50-60 spreads were analysed per condition.

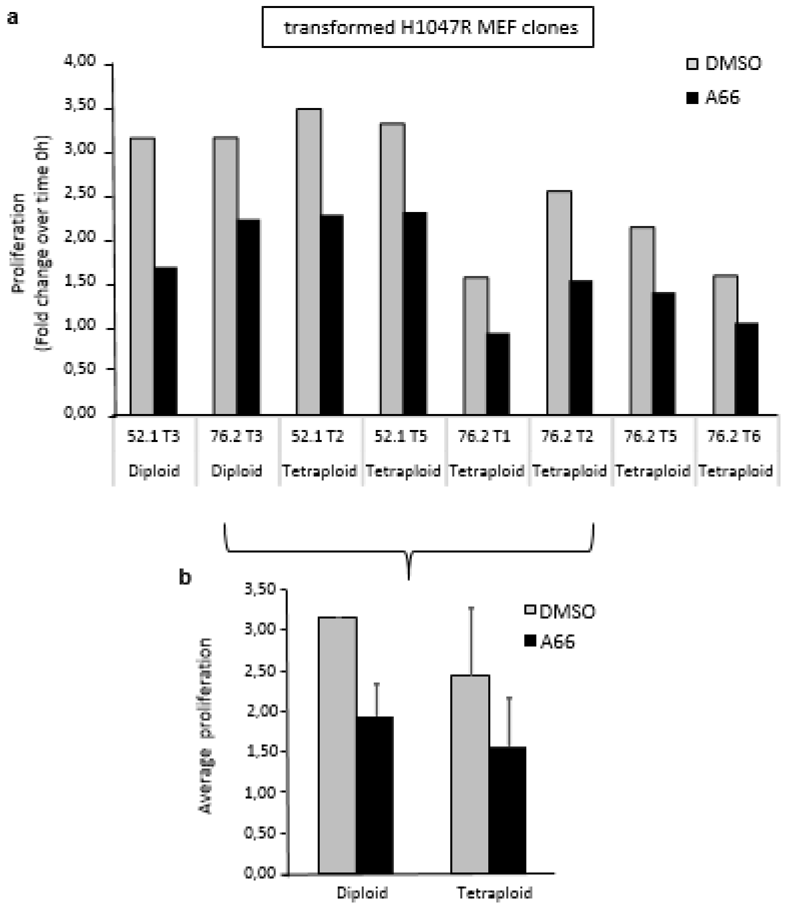

Supplementary Figure 18: Sensitivity of diploid and tetraploid transformed p110α^{H1047R} MEFs to p110α inhibition. **a**) Impact of inhibition of p110α on the proliferation of transformed diploid and tetraploid transformed p110α^{H1047R} MEFs. One experiment using 2 diploid clones and 6 diploid clones was performed. Cells were treated for 3 days with vehicle or A66 (3 µM), followed by staining with MTS. **b**) Average of the proliferation data shown in a). Error bars indicate standard deviation of the comparison of diploid to tetraploid MEF clones.

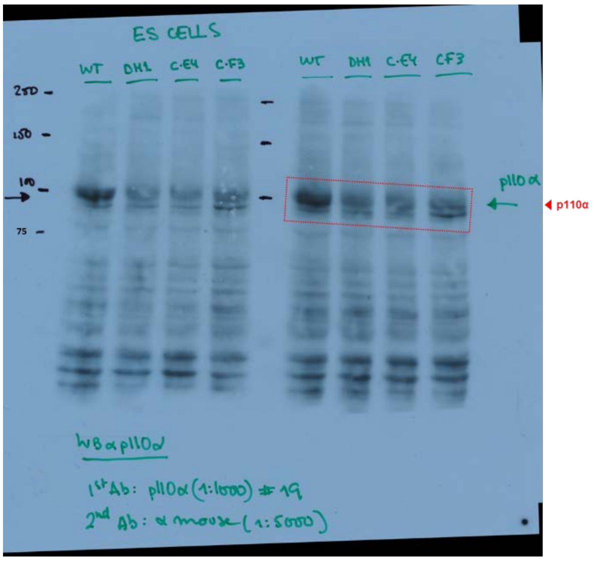

Supplementary Figure 19: related to Fig. 1b

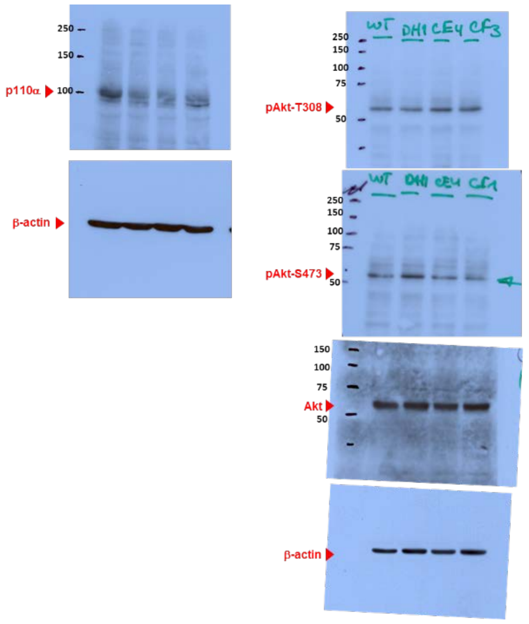

Supplementary Figure 19: related to Fig. 1b

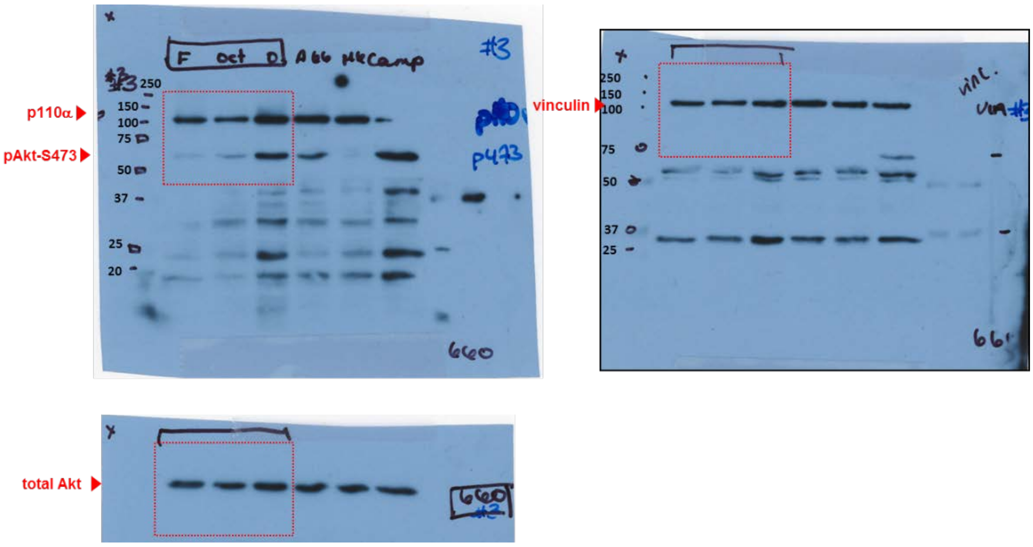

Supplementary Figure 19: Related to Fig. 1c.

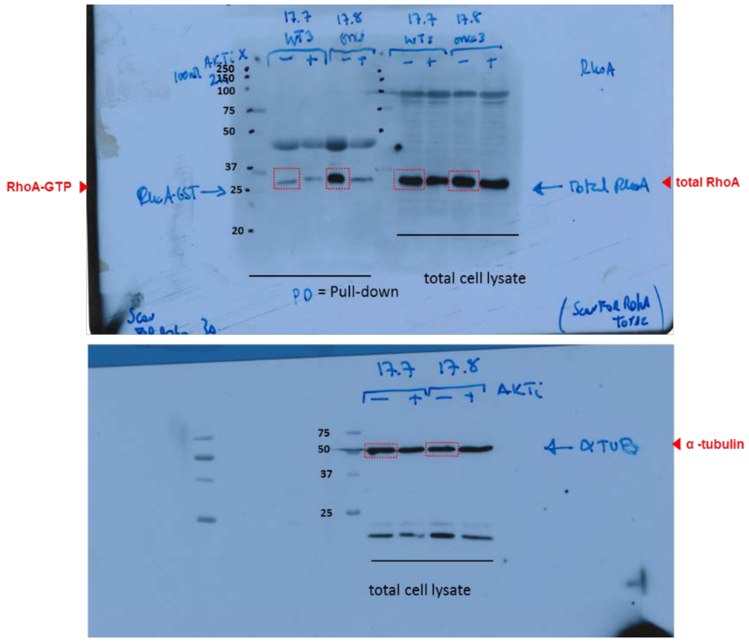

Supplementary Figure 19: Related to Fig. 4a.

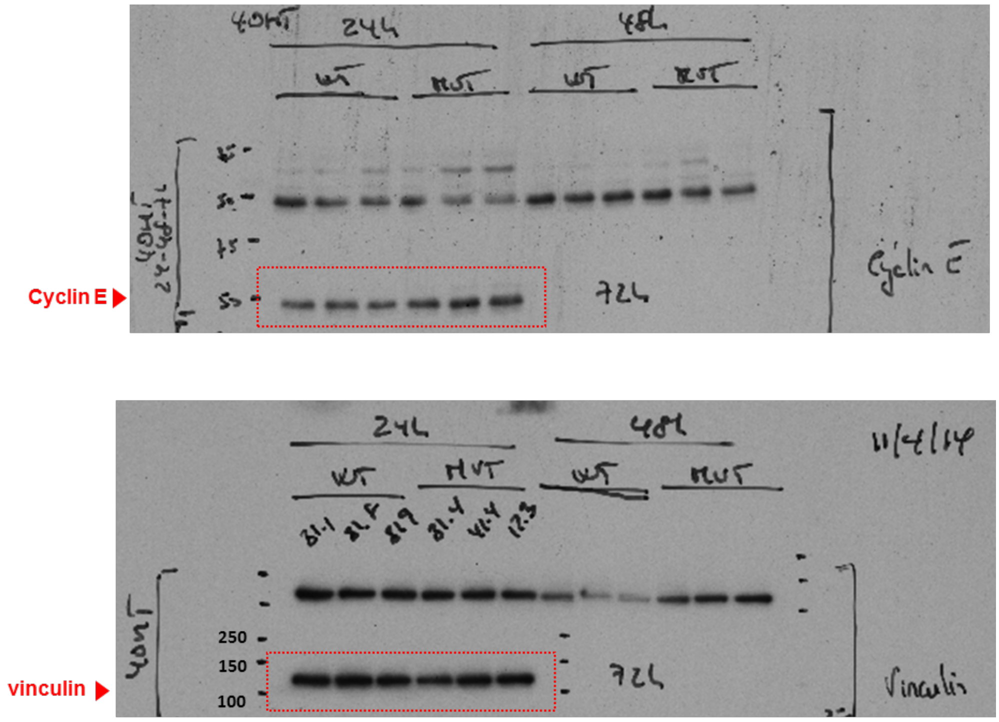

Supplementary Figure 19: related to Fig. 4b.

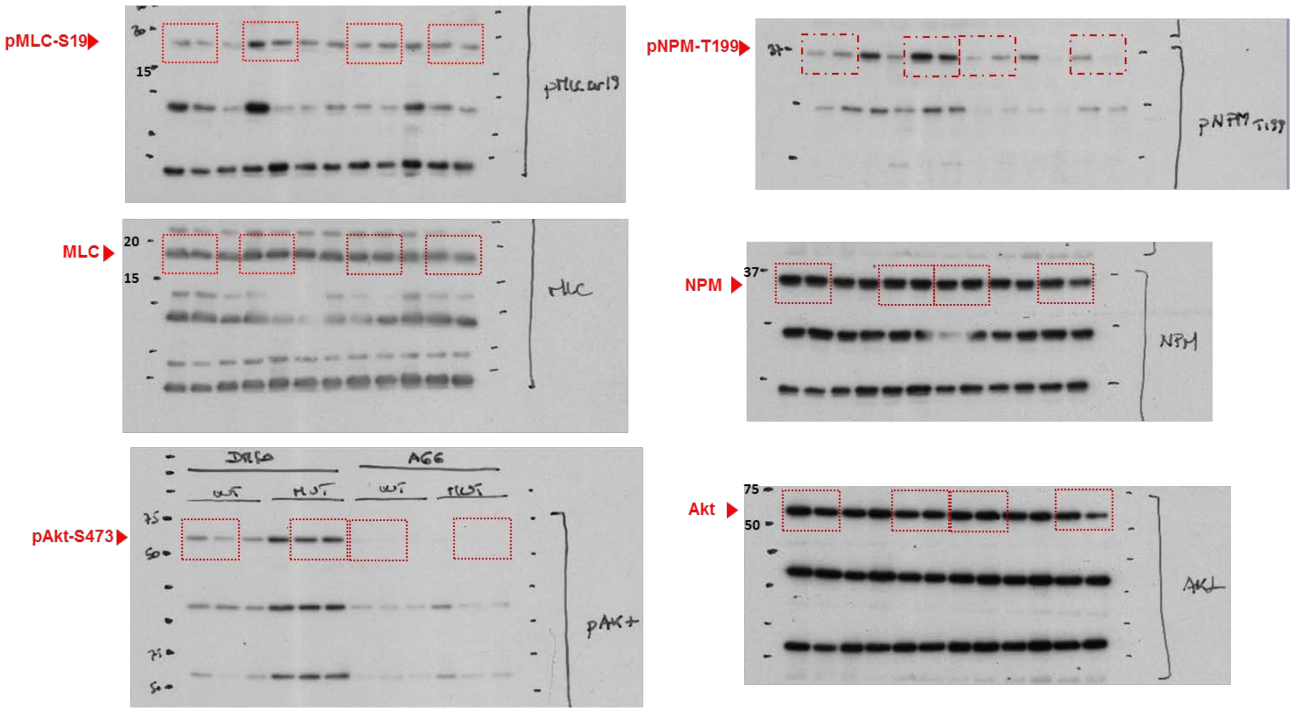

Supplementary Figure 19: related to Fig. 4c.

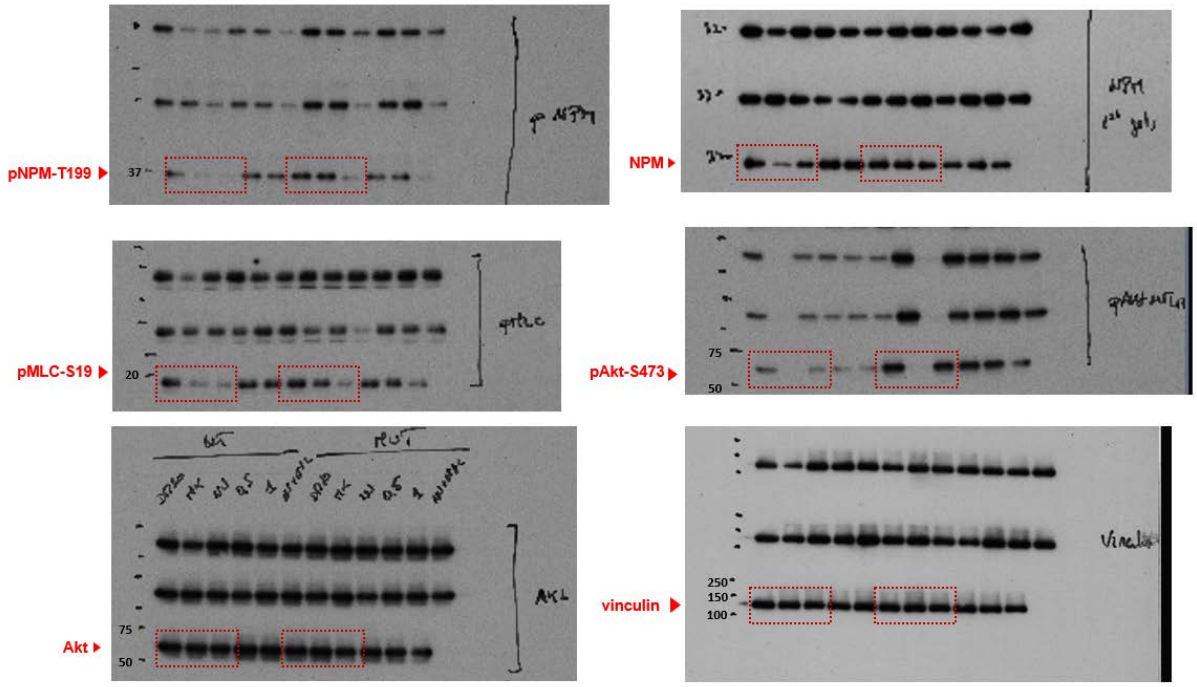

Supplementary Figure 19: related to Fig. 4d.

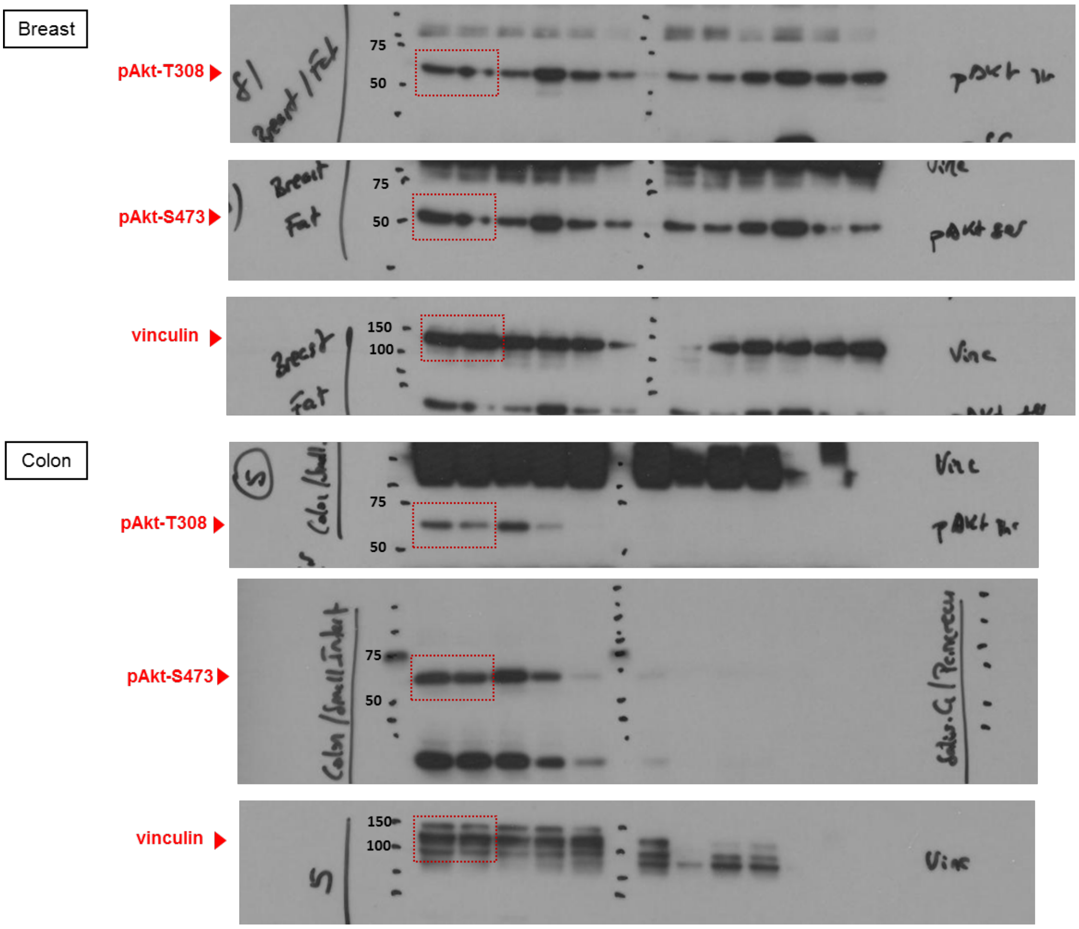

Supplementary Figure 19: related to Supplementary Fig. 1a.

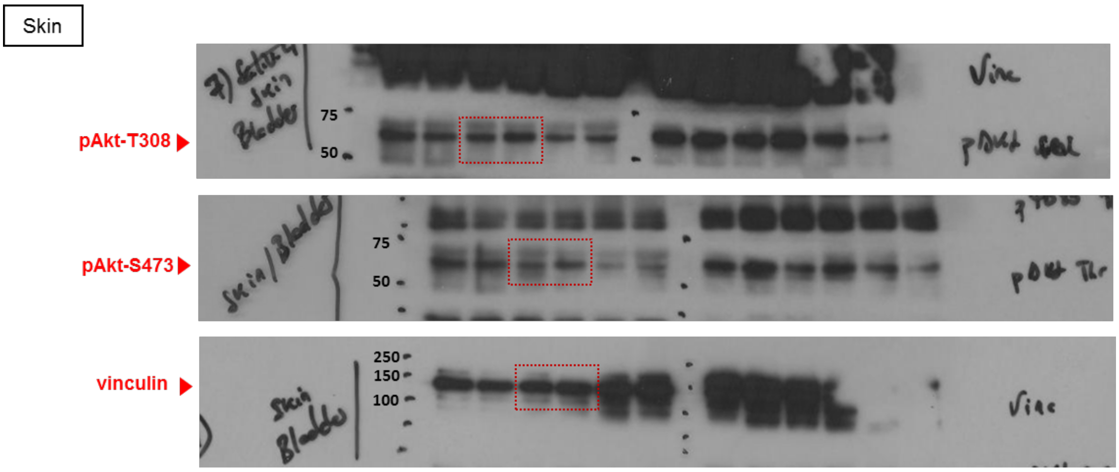

Supplementary Figure 19: related to Supplementary Fig. 1a.

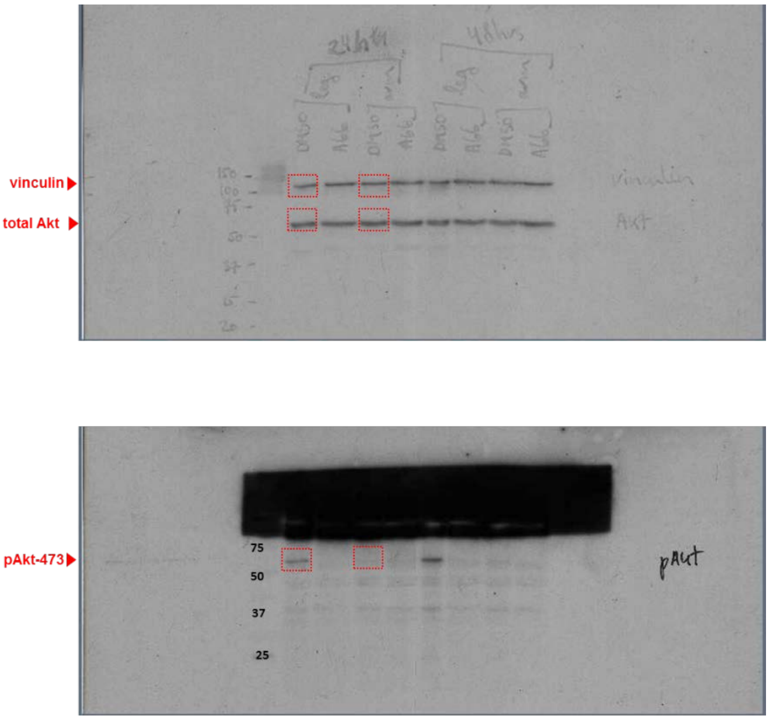

Supplementary Figure 19: Related to Supplementary Fig. 1c.

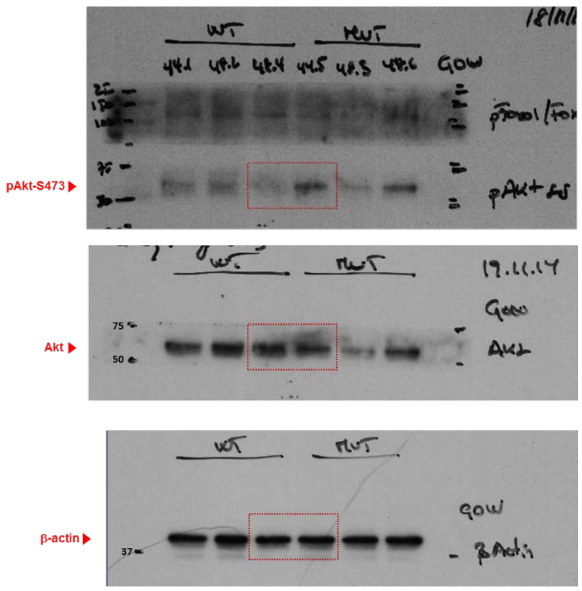

Supplementary Figure 19: related to Supplementary Fig. 2a, left panel.

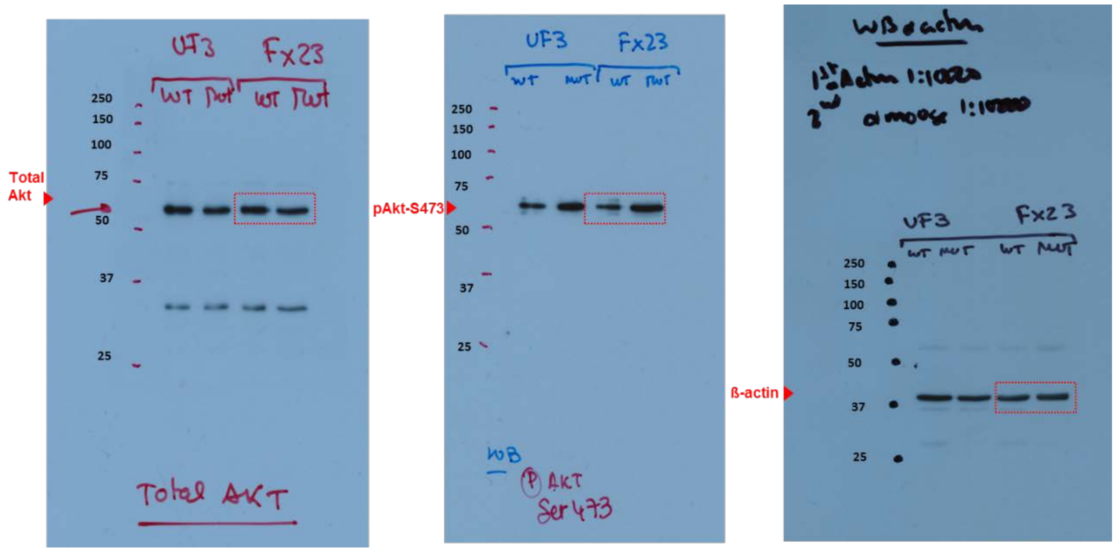

Supplementary Figure 19: related to Supplementary Fig. 2a, right panel.

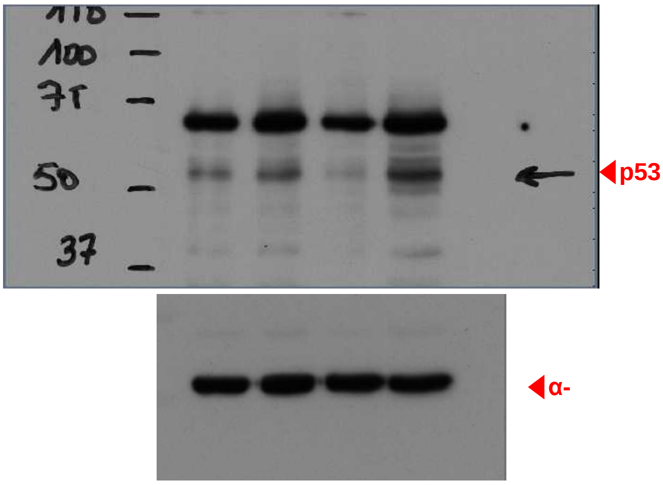

Supplementary Figure 19: related to Supplementary Fig. 2e.

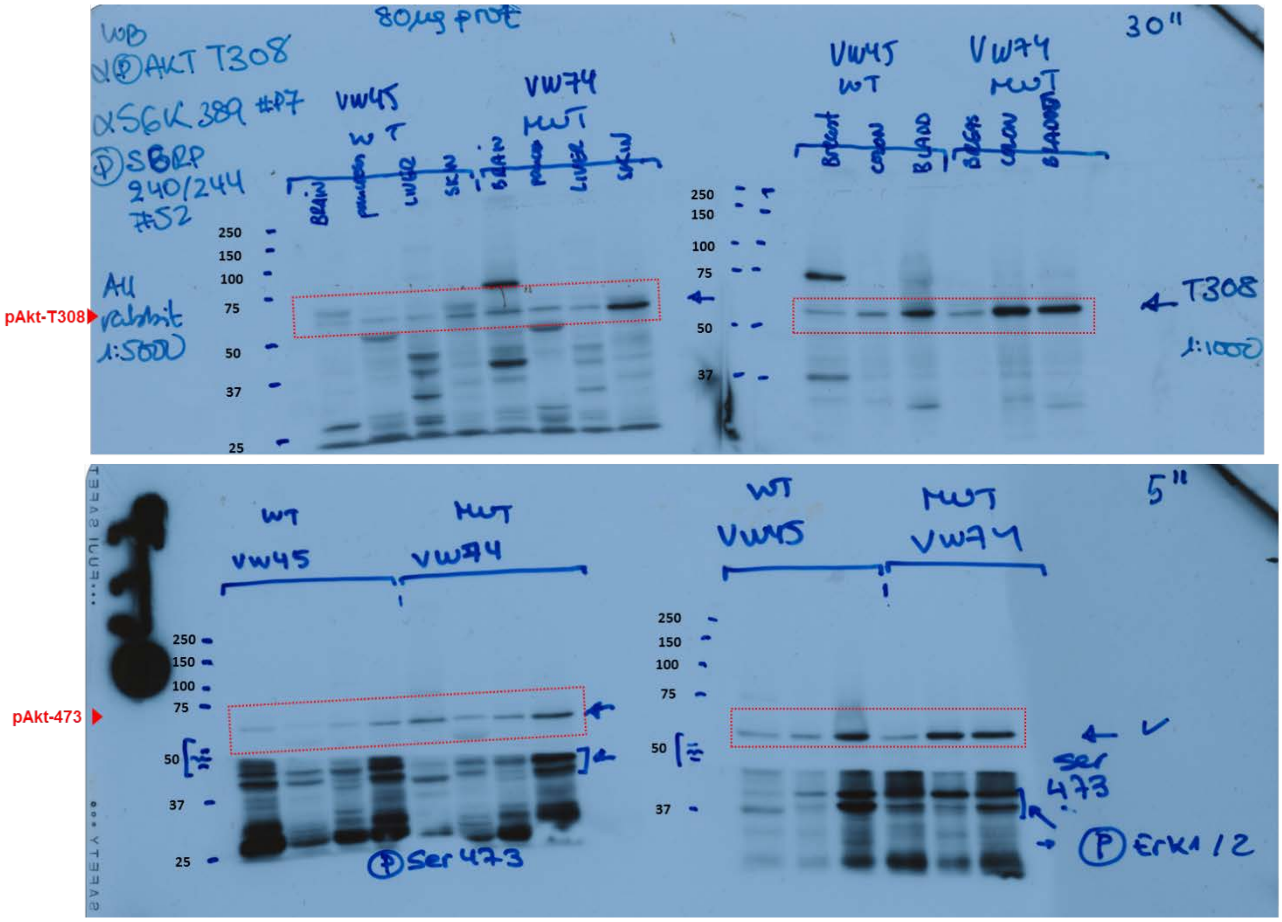

Supplementary Figure 19: related to Supplementary Fig. 3b.

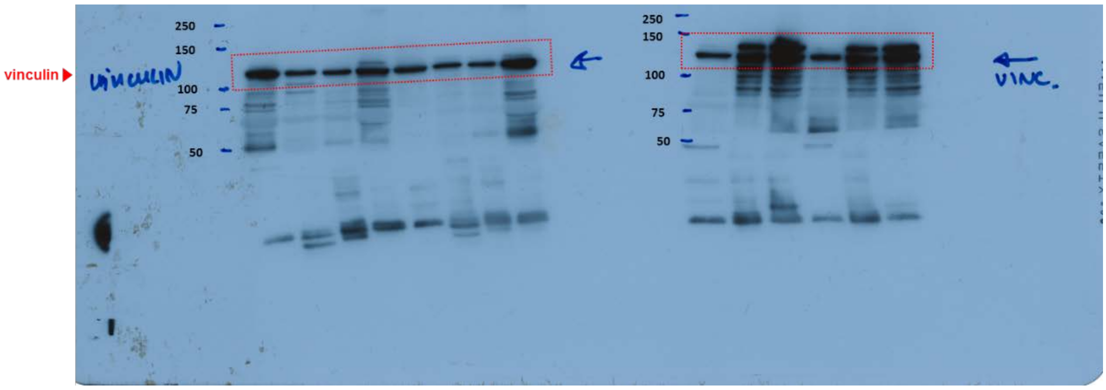

Supplementary Figure 19: related to Supplementary Fig. 3b.

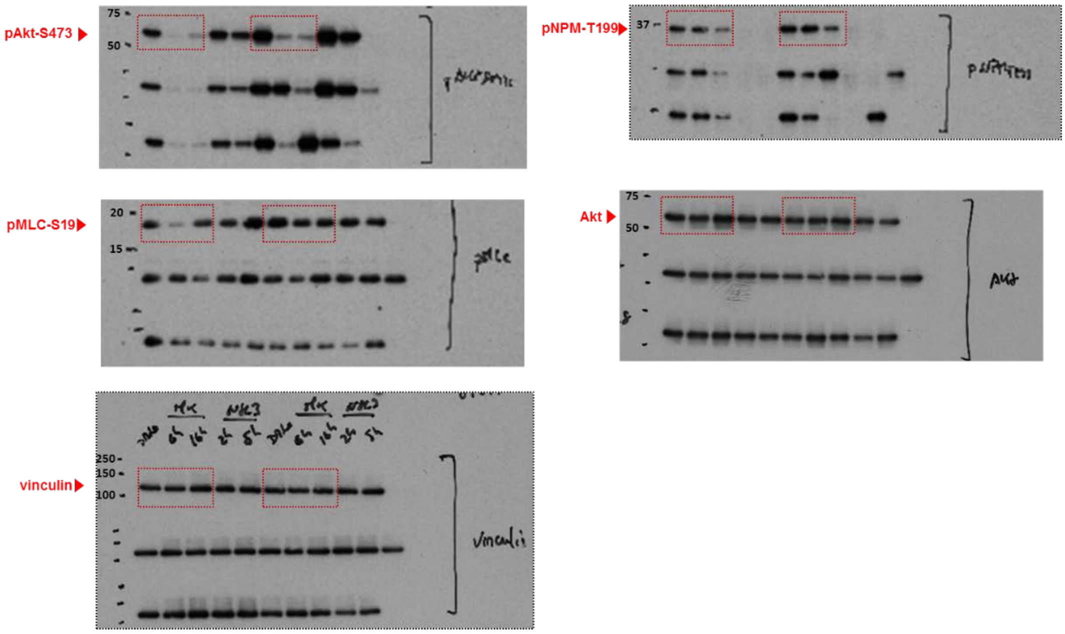

Supplementary Figure 19: related to Supplementary Fig. 8a.

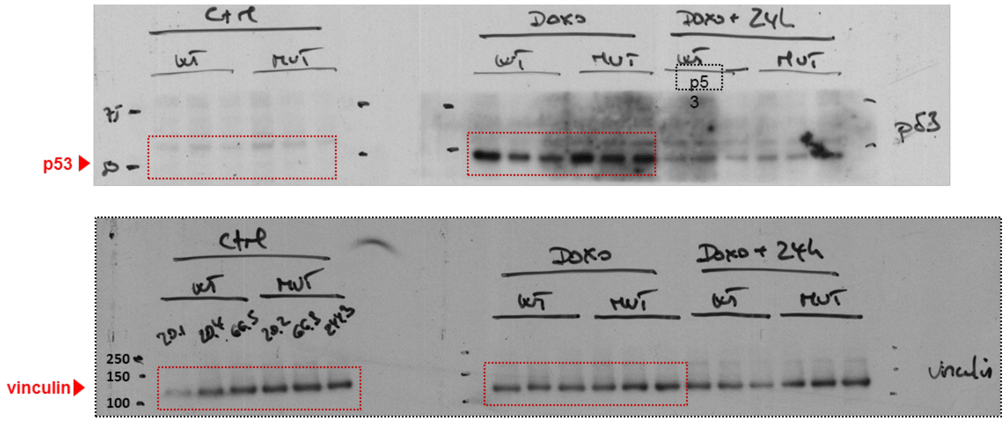

Supplementary Figure 19: related to Supplementary Fig. 14b.

Supplementary Table 1: List of H&E-stained organs and tissues subjected to histological examination.

adrenal	perigenital fat pad
brain	perirenal fat pad
colon	preputial gland
eyes	prostate
gall bladder	rectum
harderian gland	salivary gland
heart	skin
kidney	spleen
liver	stomach
lungs	testis/ovaries
mammary glands	thymus
skeletal muscle	thyroid
optical nerve	urinary bladder
pancreas	vagina/uterus

Supplementary Table 2: Cohort of mice treated with tamoxifen[1] and age at which they were subjected to histological examination by H&E staining.

Mouse ID (1)	Sex	Genotype	Age (weeks)
CXZ7.1	f	$Pik3ca^{WT};Flpe\text{-}ER^{T2}$	10.6
XV58	f	$Pik3ca^{WT};Flpe\text{-}ER^{T2}$	17.1
XV17	f	$Pik3ca^{WT};Flpe\text{-}ER^{T2}$	20.6
XV13	f	$Pik3ca^{WT};Flpe\text{-}ER^{T2}$	30.8
XV4	f	$Pik3ca^{WT};Flpe\text{-}ER^{T2}$	41.4
VW45	f	$Pik3ca^{WT};Flpe\text{-}ER^{T2}$	49
XV36	m	$Pik3ca^{WT};Flpe\text{-}ER^{T2}$	14.3
VW77	m	$Pik3ca^{WT};Flpe\text{-}ER^{T2}$	15.3
XV20	m	$Pik3ca^{WT};Flpe\text{-}ER^{T2}$	20.6
XV27	m	$Pik3ca^{WT};Flpe\text{-}ER^{T2}$	28.4
XV33	m	$Pik3ca^{WT};Flpe\text{-}ER^{T2}$	37.5
XV28	m	$Pik3ca^{WT};Flpe\text{-}ER^{T2}$	40.3
XV12	m	$Pik3ca^{WT};Flpe\text{-}ER^{T2}$	42.9
VW66	m	$Pik3ca^{WT};Flpe\text{-}ER^{T2}$	42.9
VW72	f	$Pik3ca^{H1047R+neo};Flpe\text{-}ER^{T2}$	11.2
XV60	f	$Pik3ca^{H1047R+neo};Flpe\text{-}ER^{T2}$	17.1
XV26	f	$Pik3ca^{H1047R+neo};Flpe\text{-}ER^{T2}$	28.4
XV14	f	$Pik3ca^{H1047R+neo};Flpe\text{-}ER^{T2}$	30.8
VW74	f	$Pik3ca^{H1047R+neo};Flpe\text{-}ER^{T2}$	38.1
XV5	f	$Pik3ca^{H1047R+neo};Flpe\text{-}ER^{T2}$	42.9
VW59	f	$Pik3ca^{H1047R+neo};Flpe\text{-}ER^{T2}$	43.9
XV69	f	$Pik3ca^{H1047R+neo};Flpe\text{-}ER^{T2}$	62.5
XV34	m	$Pik3ca^{H1047R+neo};Flpe\text{-}ER^{T2}$	37.5
VW64	m	$Pik3ca^{H1047R+neo};Flpe\text{-}ER^{T2}$	42.9
VW65	m	$Pik3ca^{H1047R+neo};Flpe\text{-}ER^{T2}$	42.9

[1]This is the mouse cohort shown in Supplementary Fig. 3c,d.
