# Supplementary material for: Oncogenic PIK3CA induces centrosome amplification and tolerance to genome doubling
Source: Nat Commun. 2017 Nov 24;8:1773. doi: 10.1038/s41467-017-02002-4 (PMC5701070; doi:10.1038/s41467-017-02002-4)
Supplement: Supplementary file 3 — Description of Additional Supplementary Files [file 41467_2017_2002_MOESM3_ESM.pdf]

Description of Additional Supplementary Files

File Name: Supplementary Movie 1

Description: Efficient mitosis and cell division in p110αH1047R MEFs with centrosome amplification. Filming of Cent2-GFP;Pik3caH1047R+neo;Flpe-ERT2 MEFs started 48 h after 4-OHT treatment and for a period of 30 h.

File Name: Supplementary Movie 2

Description: Efficient mitosis and cell division in p110αH1047R MEFs with centrosome amplification. Filming of Cent2-GFP;Pik3caH1047R+neo;Flpe-ERT2 MEFs started 48 h after 4-OHT treatment and for a period of 30 h.

File Name: Supplementary Movie 3

Description: Efficient mitosis and cell division in p110αH1047R MEFs with centrosome amplification. Filming of Cent2-GFP;Pik3caH1047R+neo;Flpe-ERT2 MEFs started 48 h after 4-OHT treatment and for a period of 30 h.

File Name: Supplementary Movie 4

Description: Representative video of p110αH1047R binucleated MEFs dividing after DCB washout. Filming of Pik3caH1047R+neo;Flpe-ERT2 MEFs started 72 h after 4-OHT treatment and for a period of 30 h.
